# What fragile factors hinder the pace of China’s alleviation efforts of the poverty-stricken population? A study from the perspective of impoverishment caused by medical expenses

**DOI:** 10.1186/s12913-022-08237-2

**Published:** 2022-07-29

**Authors:** Jiahui Wang, Xinye Qi, Linghan Shan, Kexin Wang, Xiao Tan, Zheng Kang, Ning Ning, Libo Liang, Lijun Gao, Mingli Jiao, Yu Cui, Yanhua Hao, Qunhong Wu, Ye Li

**Affiliations:** 1 Centre of Health Policy & Management, Health Management College, Harbin Medical University, 157 Baojian Road, Nangang District, Harbin, 150086 Heilongjiang China; 2 Department of Social Medicine, School of Public Health, Health Management College, Harbin Medical University, No.157 Baojian Road, Nangang District, Harbin, 150086 Heilongjiang China; 3 Shenzhen Hospital of Guangzhou University of traditional Chinese Medicine (Futian), 6001 Beihuan Avenue, Futian District, Shenzhen, Guangdong Province China

**Keywords:** Medical impoverishment, Poverty alleviation, Financial protection, China

## Abstract

**Objective:**

China has made remarkable achievements in poverty alleviation. However, with the change in economic development and age structure, the population stricken by poverty due to medical expenses and disability accounted for 42.3 and 14.4% of the total poverty-stricken population, respectively. Accordingly, it is crucial to accurately pinpoint the characteristics of people who are about to become poor due to illness. In this study, we analyzed the incidence of impoverishment by medical expense at the provincial, family, and different medical insurance scheme levels to identify the precise groups that are vulnerable to medical-related poverty.

**Method:**

Data were extracted from the Fifth National Health Service Survey in China in 2013 through a multi-stage, stratified, and random sampling method, leaving 93,570 households (273,626 people) for the final sample. The method recommended by World Health Organization (WHO) was adopted to calculate impoverishment by medical expense, and logistic regression was adopted to evaluate its determinants.

**Results:**

The poverty and impoverishment rate in China were 16.2 and 6.3% respectively. The poverty rate in western region was much higher than that of central and eastern regions. The rate of impoverishment by medical expense (IME) was higher in the western region (7.2%) than that in the central (6.5%) and eastern (5.1%) regions. The New Cooperative Medical Scheme (NCMS) was associated with the highest rate (9.1%) of IME cases. The top three diseases associated with IME were malignant tumor, congenital heart disease, and mental disease. Households with non-communicable disease members or hospitalized members had a higher risk on IME. NCMS-enrolled, poorer households were more likely to suffer from IME.

**Conclusion:**

The joint roles of economic development, health service utilization, and welfare policies result in medical impoverishment for different regions. Poverty and health service utilization are indicative of households with high incidence of medical impoverishment. Chronic diseases lead to medical impoverishment. The inequity existing in different medical insurance schemes leads to different degrees of risk of IME. A combined strategy to precise target multiple vulnerabilities of poor population would be more effective.

**Supplementary Information:**

The online version contains supplementary material available at 10.1186/s12913-022-08237-2.

## Introduction

China has made remarkable achievements in poverty alleviation and the incidence of poverty in the rural population has drastically dropped from 10.2% in 2012 to 0.6% in 2019 [1]. The current poverty alleviation achievements solve the overall regional poverty of the rural poor based on a certain standard of absolute poverty. In the definition of absolute poverty, minimum living needs or basic needs are the core, but there are differences in the definition of basic needs between living needs (clothing, food, housing, and transportation, etc.), social participation needs, and social security needs (education, culture, and public environmental sanitation, etc.) [2, 3]. The concept of absolute poverty, widely accepted in China, is a situation in which individuals or families cannot maintain their basic survival needs depending on their labor income and other legal income [4, 5]. Moreover, according to basic living food expenditure and non-food expenditure, the rural income poverty line was set as three standards in 1978, 2008 and 2010, respectively [6]. However, the elimination of absolute poverty measured by a single income does not mean the disappearance of poverty, it just shows that the measure of absolute poverty is no longer applicable [7]. It is worth noting that although the income poverty line is used to measure poverty in China, poverty alleviation policies have not been limited to increasing the income of the poor since the beginning of reform and opening up and have penetrated from multiple dimensions such as education, health, and culture.

Multi-dimensional poverty is more serious than income poverty alone in China due to imbalances in the aspects of age, economy, and geography. Specifically, the incidence of poverty due to education, disease, and so on are higher than those due to low income, and poor health is considered to be the greatest factor pushing vulnerable households into poverty [8]. Some data showed that the population stricken by poverty due to medical expenses and disability accounted for 42.3 and 14.4% of the total poverty-stricken population, respectively [9]. Therefore, China has adopted a series of health poverty alleviation policies, which manifest that the government and relevant departments aim at improving the medical and health service capacity, medical security level and public health service level for the poor people or people in poverty-stricken areas, and ultimately improving their health conditions, and solving the problems of poverty caused by illness and returning to poverty due to illness. China has made great efforts in medical services, medical insurance and public health. Of which, research on medical expenses, especially catastrophic health expenditures, plays an important role in both “health poverty alleviation” and “targeted approach to alleviating poverty”.

However, with the age structure of chronic diseases showing a clear younger trend in recent years, more and more middle-aged people are trapped in poverty due to the economic burden of long-term treatment of these diseases, especially for those with relatively low economic status [10]. Therefore, the study of the group of vulnerable people who do not belong to the reported poverty-stricken population is very important.

Previous studies mostly focused on the incidence and determinants of IME, Sanjay K estimated the IME incidence in India from 2014 to 2018 and found that the indicator declined from 5.1 to 3.3% [11], a study from Ethiopian showed that households living in urban areas are more likely to drop into IME, but households headed by males and with formal education are protected factors for IME [12]. Sayem reported the incidence of IME in Bangladesh was 4.5% and utilization of private facilities, having elderly people, chronic illness and location were risk factors [13]. A study from Korea identified the causal effect of health shocks on poverty status and explored the mechanisms of IME, which indicated that IME is impacted by health shocks through the labor force [14]. Zhao estimated the IME incidence and the socio-economic disparities trend from 2011 to 2016 and found that the value decreased from 7.34% to 5.14 and the decrease in level of impoverishment was less in rural areas [15]. Xu [16] and Fu [17] explored the effect of New Health Care Reform and cancer treatment on IME and provided an in-depth analysis of explanatory variables. However, studies tend to lack the consideration of more comprehensive factors and a more in-depth analysis of multiple characteristics.

Poverty alleviation efforts might focus on not only the people who are currently poor but also the increase in poverty that may occur. Accordingly, it is crucial to accurately pinpoint the characteristics of people who are about to become poor due to illness. We analyzed the incidence of impoverishment by medical expense (IME) at the provincial, family, and different medical insurance scheme levels to identify the precise groups that are vulnerable to medical-related poverty and wish to provide a reference of relative poverty standard for the “post-poverty era” and to serve the establishment of a long-term mechanism.

## Method

### Study design

This is a cross-sectional study on incidence of poverty and impoverishment by medical expense, households with the highest risk of impoverishment from aspect of household head, household and healthcare needs, and healthcare utilization would be focused on.

### Data source and sampling methods

**Fig. 1:**
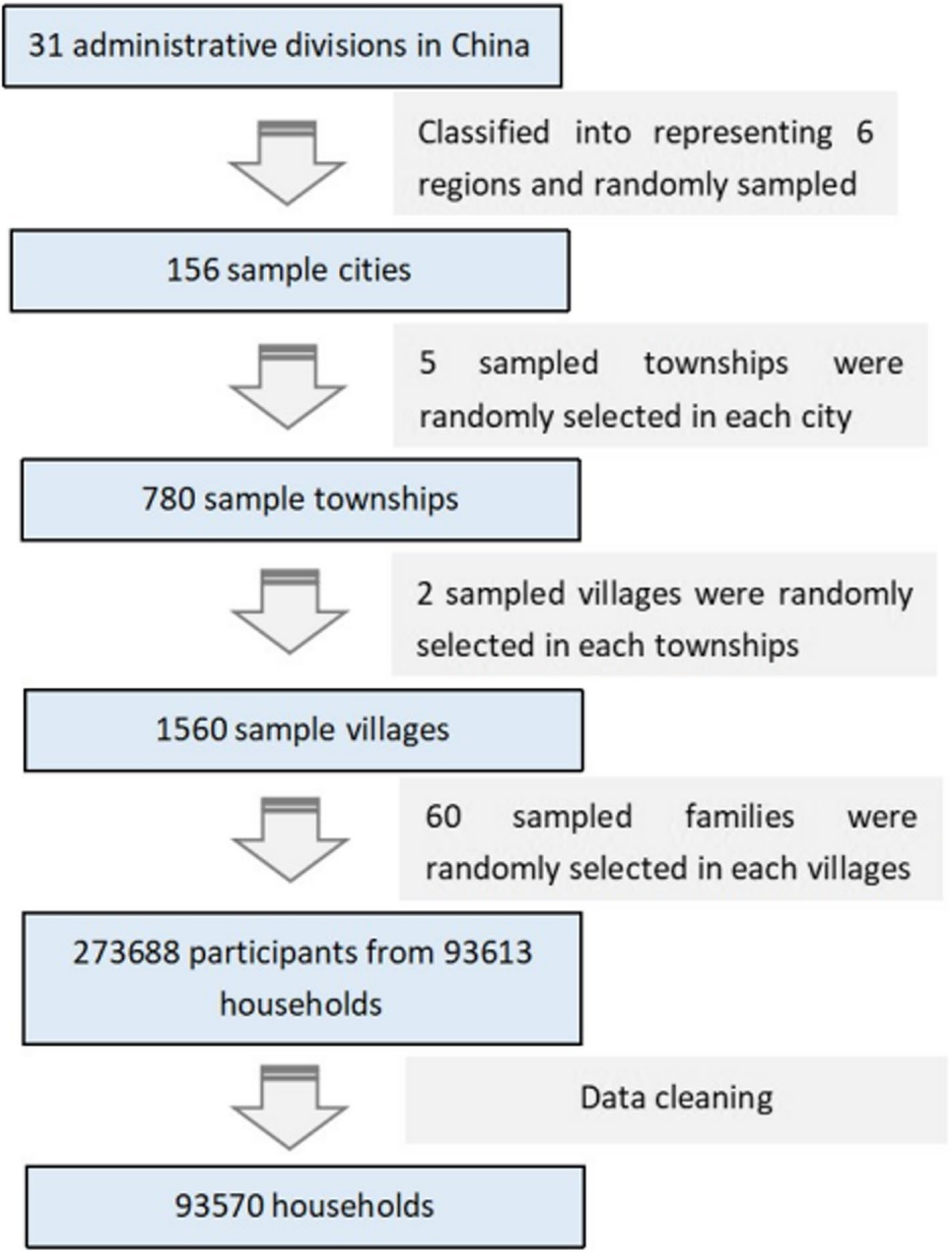
The detail sampling process of the study

The data we used were from the Fifth National Health Service Survey (NHSS) in China, 2013. This survey is conducted every five years and has the most nationally representative sample obtained through a multi-stage, stratified, and random sampling method. Finally, 31 provinces, 156 districts (or counties), 780 townships, 1560 villages, and 93,613 households (273,688 people) were included in the survey [18], after data cleaning, 93,570 households were included in this study, the detailed sampling process was shown in Fig. 1.

### Data collection and quality control

Face-to-face interviews were conducted to collect the information of participants, including the households’ basic information, healthcare needs, and healthcare utilization. Data quality was strictly controlled during the whole survey and investigation process. Every investigator received rigorous training before the investigation. To ensure the effectiveness of the survey, a subset of the respondents was selected to answer the questionnaire again, reaching a consistency rate of 97.7%. Based on data from the sixth census in 2010, an analysis using the Maria index, delta dissimilarity coefficient, GINI concentration ratio, and fitting degree test showed that the size of the sample family and the proportion of rural versus urban households were not different from that of the whole country, but the proportion of the elderly population was higher than that of the general population [18].

### Variables

#### Dependent variable

The dependent variable of this study is whether a family experienced impoverishment due to medical expenses or not (no = 0, yes = 1) and whether a family was in poverty (Yes = 1, No = 0).

#### Independent variable

Based on existing literature, we chose household head and household level indicators as independent variables, including demographic characteristics of household and household head, healthcare needs and health service utilization items, Table 1 showed the codes of the variables. The household head variable included the household head’s gender (Female = 0, Male = 1), education level (Illiterate = 1, Primary school = 2, Junior high school = 3, Senior high school & & technical school & technical secondary school = 4, Junior college and above = 5), marital status (Married = 0, Others = 1), employment status (Employed = 1, Retired = 2, Unemployed and students = 3), and insurance scheme type (Medical Insurance for Urban Employees Scheme, UE-BMI = 1, Urban Resident Basic Medical Insurance, UR-BMI = 2, New Cooperative Medical Scheme, NCMS = 3, Integrating Basic Medical Insurance for Urban and Rural Residents, IBMIUR = 4, Mixture of schemes = 5, Other types and none = 6). The household-level variable included region (East = 1, Middle = 2, West = 3), residential area (Urban = 0, Rural = 1), economic level (Quintile 1 = 1, Quintile 2 = 2, Quintile 3 = 3, Quintile 4 = 4, Quintile 5 = 5), household size (≤2 = 1, 3–4 = 2, ≥ 5 = 3), households with members over 60 years old (No = 0, Yes = 1) or younger than five years old (No = 0, Yes = 1), households with non-communicable disease (NCD) members, with inpatient members (0 = 1, 1 = 2, ≥2 = 3). Preferred institution grade for common diseases (0 = Primary hospital, 1 = Non-primary hospital), whether there is a member going to a clinic in 2 weeks (No = 0, Yes = 1), member not seeing a doctor/hospitalized despite the need for it (No = 0, Yes = 1).

**Table 1:** The basic information of the respondents

Variables	Percentage (%)
**Economics quintile**	
Quintile 1	18,714 (20.00)
Quintile 2	18,714 (20.00)
Quintile 3	18,714 (20.00)
Quintile 4	18,714 (20.00)
Quintile 5	18,714 (20.00)
**Household size**	
≤ 2	41,633 (44.50)
3–4	39,289 (42.00)
≥ 5	12,648 (13.50)
**Region**	
Eastern	31,201 (33.30)
Central	31,186 (33.30)
Western	31,183 (33.30)
**Location**	
Urban	46,798 (50.00)
Rural	46,772 (50.00)
**Gender**	
Male	69,826 (74.60)
Female	23,744 (25.40)
**Marital status of household head**	
Married	78,751 (84.20)
Others	14,819 (15.80)
**Educational level of the head of household**	
Illiterate	9904 (10.60)
Primary school	26,556 (28.40)
Junior high school	33,742 (36.10)
Senior high school & & technical school& technical secondary school	11,800 (12.60)
Junior college and above	11,568 (12.40)
**Employment status of the head of household**	
Employed	64,026 (68.40)
Retired	16,164 (17.30)
unemployed and students	13,380 (14.30)
**Medical insurance of the head of household**	
UE-BMI	23,919 (25.60)
UR-BMI	6847 (7.30)
NCMS	43,362 (46.30)
IBMIUR	12,060 (12.90)
Mixture of schemes	4811 (5.1%)
Other types and none	2571 (2.7%)
**Households including members aged above sixty years**	
No	52,792 (56.40)
Yes	40,778 (43.60)
**Households including members aged below five years**	
No	77,239 (82.50)
Yes	16,331 (17.50)
**Number of patients with chronic diseases**	
0	52,793 (56.40)
1	30,316 (32.40)
≥ 2	10,461 (11.20)
**Number of hospitalized members**	
0	74,641 (79.80)
1	16,795 (17.90)
≥ 2	2134 (2.30)
**Preferred institution grade for common diseases**	
Primary hospital	75,833 (81.00)
Non-primary hospital	17,737 (19.00)
**Whether there is a member go to clinic**	
No	75,006 (80.20)
Yes	18,564 (19.80)
**Members should be hospitalized but not**	
No	89,516 (95.70)
Yes	4054 (4.30)
**Members should see a doctor but not**	
No	66,440 (71.00)
Yes	27,130 (29.00)

*Notes*: *UE-BMI* Medical Insurance for Urban Employees Scheme, *UR-BMI* Medical Insurance for Urban Residents Scheme, *NCMS* New Cooperative Medical Scheme, *IBMIUR* Integrating Basic Medical Insurance for Urban and Rural Residents; Mixture of schemes means people are enrolled in commercial medical insurance and social medical insurance at the same time

### Measurement

#### Poverty and IME

The method recommended by the World Health Organization (WHO) was adopted in this study [19]. Poverty was defined if the total expenditure of a household was less than the subsistence spending of each household. Impoverishment by medical expense was defined as non-poor households falling into poverty owing to health service payments. The calculation progress of related key indicators is as follows:

Equivalent family size:

1 $${eqsize}_h={hhsize_h}^{0.56}$$

Equivalent food expenditure:

2 $${eqfood}_h=\frac{food_h}{eqsize_h}$$

Equivalent food expenditure is total household food expenditure divided by equivalent family size, *food*_*h*_ is food expenditure of each household, *eqsize*_*h*_ is equivalent family size.

Poverty Line (PL):

3 $$\frac{\sum {w}_h\ast {eqfood}_h}{\sum {w}_h}$$

Poverty Line refers to the weighted average annual food expenditure of a household whose food expenditure share of total household consumption expenditure is between the 45th and 55th percentiles of the entire sample.

Household subsistence expenditure (SE):

4 $${se}_h= pl\ast {eqsize}_h$$

The definition of poverty household:

5 $$if\ poor=1\ {\mathit{\exp}}_h<{se}_h$$

*exp*_*h*_ is total household consumption expenditure, when the total household consumption expenditure is less than its subsistence expenditure, vice versa.

The definition of a household getting impoverished due to medical expense:

6 $$If\ {impoor}_h=1\ {\mathit{\exp}}_h\ge {se}_h>{\mathit{\exp}}_h-{oop}_h$$

*oop*_*h*_ refers to out-of-pocket medical payment (OOP)_*,*_ when the total household consumption expenditure is more than subsistence expenditure but less than less than subsistence spending after health out-of-pocket payment, the household was categorized as impoverished*.*

### Data analysis

The chi-square test was used for univariate analyses to identify factors associated with medical impoverishment and logistic regression was adopted to select the determinants. All statistical analyses were performed using STATA 11.0. *P*-values < 0.05 were considered statistically significant.

## Results

### Basic information of respondents

Most householders were male (74.60%), employed (68.40%) and married (84.20%). 46.9% of households were covered by NCMS, the proportion of households with under 5 years old members and over 60 years old were 17.5 and 43.6% respectively. Households had at least 1 NCD number occupied 43.6%, the proportion of households with 1 in-patient member or 2 and above in-patient members were 17.9 and 2.3%, the detailed information is shown in Table 1.

### The rate of poverty and IME in different provinces

**Fig. 2:**
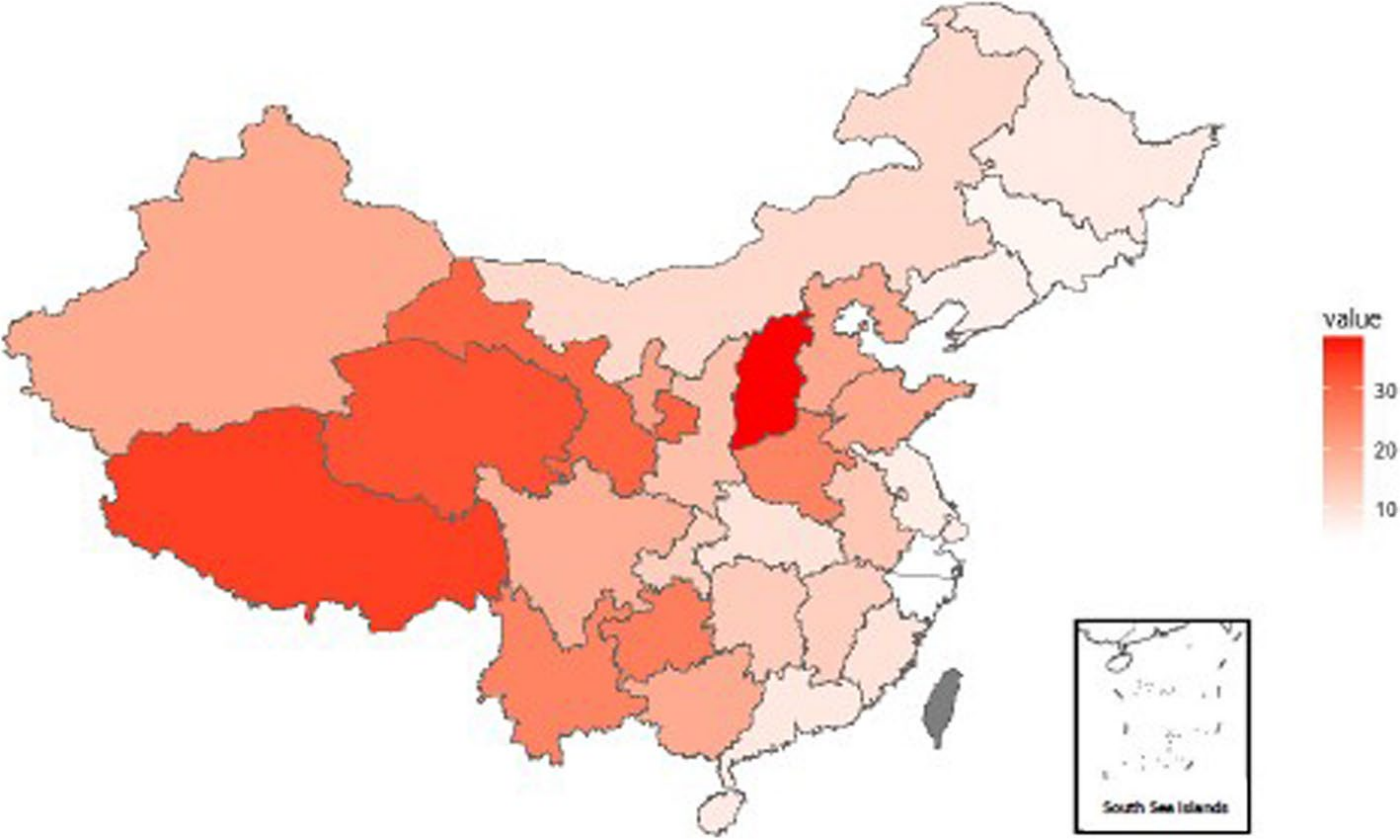
The rate of poverty in China’s 31 provinces in 2013

**Fig. 3:**
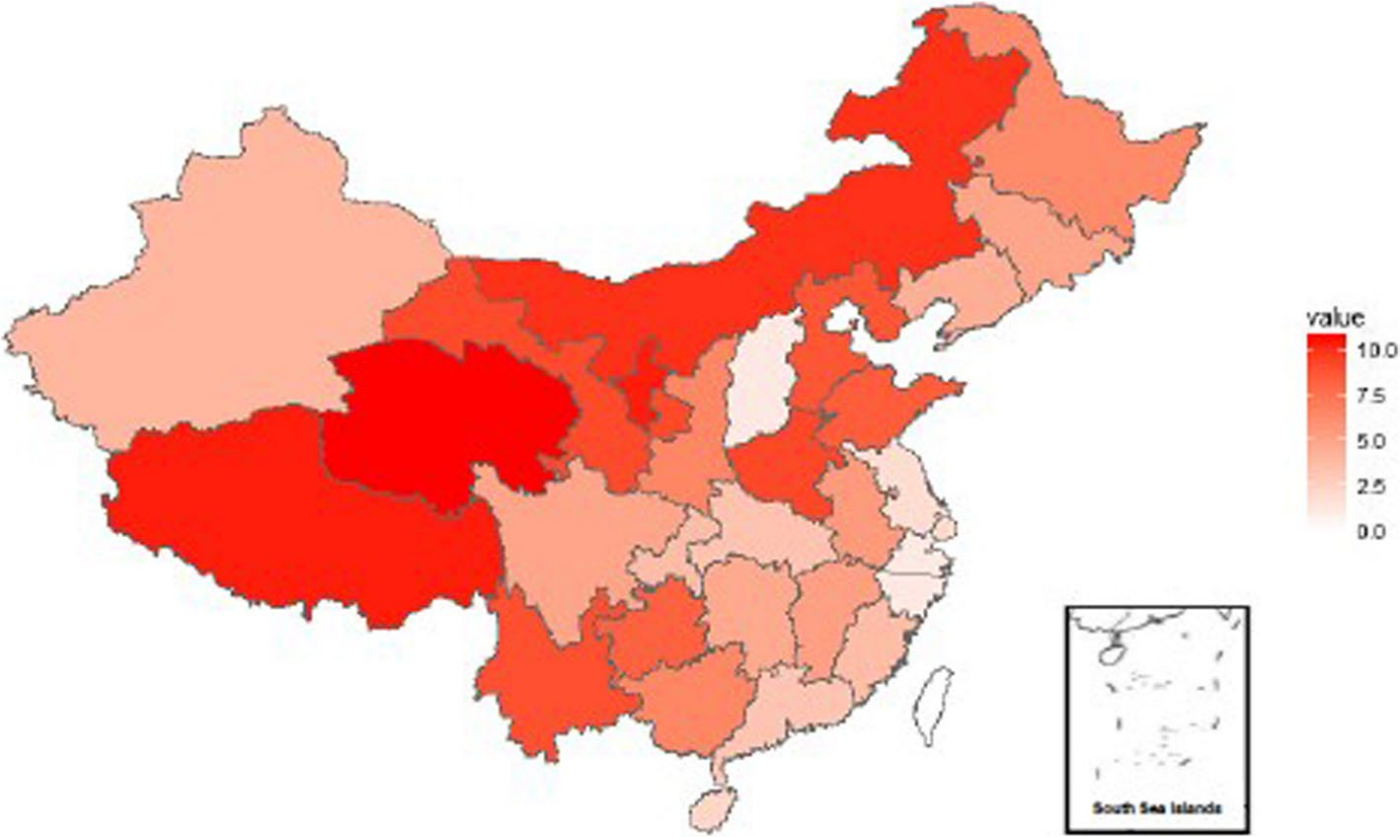
The rate of medical impoverishment in China’s 31 provinces in 2013

The poverty and IME rates in China were 16.2 and 6.3% respectively, the rates of poverty and IME in 31 provinces are shown in Fig. 2 and Fig. 3. The poverty and IME rates in China’s 31 provinces differed significantly (*P* < 0.05). Those in the western region had much higher poverty rates than the other two regions, and the value in the eastern region was the lowest. The contribution of IME to overall poverty varied widely across the 31 provinces. In some provinces, such as Inner Mongolia, Shandong, and Heilongjiang, their rates of poverty were relatively low, but they suffered from a high burden of IME. For some provinces with higher poverty rates, such as Tibet and Xinjiang, the rate of IME was not so high. Overall, the rate of IME was higher in the western region (7.2%) than that in the central (6.5%) and eastern (5.1%) regions.

### The poverty and IME rates under different medical insurance schemes

The rate of IME varied among people covered by different types of medical insurance schemes. Compared with people enrolled in other types and with no insurance schemes, those enrolled in the UE-BMI and a mixture of schemes had better capacity to deal with the burden brought by diseases. On the contrary, as one of the three basic medical insurance schemes, the incidence of IME (9.1%) was highest among those covered by the NCMS, even higher than that of people who were not covered by any medical insurance (4.0%). The IME incidence of IBMIUR was also considerably high (6.3%) (Table 2).

**Table 2:** The poverty and medical impoverishment rate under different types of medical insurance systems

Medical insurance type	OOP to total monthly household consumption(%)	Poverty(%)	Impoverishment (%)
UE-BMI	11.4	1.9	1.9
UR-BMI	12.0	11.7	6.0
NCMS	13.6	26.4	9.1
IBMIUR	12.0	14.8	6.3
Mixture of schemes	9.6	5.6	3.4
Other types and none	10.0	11.8	4.0
National Average	12.4	16.2	6.3

### Single or two-dimensional household characteristics associated with the incidence of IME

We summarized the top five household characteristics associated with the highest IME incidence and found that households with inpatient members, those with NCD members, and those at a low economic level suffered the highest incidence of IME. When the risk factors were combined, the incidence of IME for these households was nearly six times than the national average, especially for the poor households with more than two inpatient members, which had the highest incidence of 35.4% (Table 3).

**Table 3:** Top five types of families with one or two characteristic(s) associated with the incidence of medical impoverishment

	Top five types of families with one characteristic associated with medical impoverishment	Incidence (%)
**1**	Family with members who should be hospitalized but were not	14.0
**2**	Family with more than two inpatient members	13.5
**3**	Family with relatively lower income	13.5
**4**	Family with one inpatient member	11.4
**5**	Family with more than two NCD members	9.5
	Top five types of families with two characteristics associated with medical impoverishment	Incidence (%)
**1**	Poor family with more than two inpatient members	35.4
**2**	Poor family with members who should be hospitalized but were not	35.1
**3**	Poor family with one inpatient member	29.4
**4**	Poor family with more than two NCD members	26.8
**5**	Poor family with a retired household head	23.3

### Top ten diseases for which household hospitalization expenses led to medical impoverishment

A comparison of the IME groups revealed that the top three diseases associated with IME were malignant tumor, congenital heart disease, and mental disease. The total hospitalization expenses for the above-mentioned diseases all exceeded 7000 Yuan, and the highest payment was up to 11,000 Yuan for malignant tumors. Among the top 10 diseases in terms of total hospitalization expense incurred by IME households, five were chronic diseases (see Table 4).

**Table 4:** Top ten diseases in terms of total hospitalization expenses

Disease	Number of households	Total hospitalization expenses (Yuan)
Malignant tumor	184	11,000
Congenital heart disease or other congenital abnormalities	7	9000
Mental disease	19	7000
Infectious disease	46	7000
Fetal or neonatal disease	7	7000
Injury and poisoning	250	6850
Benign tumors, tumors in situ	41	6400
Disease of the blood and blood forming organ	28	5950
Neurological disease	72	5240
Endocrine, nutritional metabolic diseases, or immunity-related diseases	111	5200

Univariate analysis showed that all the factors were significantly associated with suffering impoverishment. Further logistic regression analysis showed that, many factors were related to the occurrence of impoverishment due to medical expenses. Households headed by a non-married person, an unemployed person or a student or a person with a low educational level; and households of small size, with members suffering from NCDs, with members who were inpatients were all more likely to suffer from IME. In addition, the smaller the number of people suffering from NCDs or receiving medical care in the household, the less risk of IME the household had. Households covered by the UE-BMI had the strongest ability to deal with the economic risk posed by diseases. NCMS-enrolled households had greater exposure to the risk of IME, at 1.84 times higher than that of UE-BMI-enrolled households. Poorer households were 15.05 times more likely to suffer from IME than richest households (Table 5).

**Table 5:** Determinants of medical impoverishment

	OR	95%CI		***P***-value
		Lower	Upper	
**Economics quintile (**Ref**:** Quintile 5**)**				
Quintile 1	12.83	10.91	15.09	< 0.001
Quintile 2	15.05	12.85	17.63	< 0.001
Quintile 3	2.42	2.04	2.88	< 0.001
Quintile 4	1.07	0.87	1.31	0.520
**Household size (**Ref**:** ≥5**)**				
≤ 2.	2.05	1.85	2.28	< 0.001
3–4	1.31	1.19	1.45	< 0.001
**HH Marital status (**Ref**:** Married**)**				
Others	0.87	0.80	0.95	0.204
**Gender of householder (**Ref**:** Female**)**				
Male	1.05	0.974	1.131	< 0.001
**Educational level of the head of household (**Ref**:** Junior college and above**)**				
Illiterate	1.92	1.56	2.37	< 0.001
Primary school	1.83	1.50	2.24	< 0.001
Junior high school	1.76	1.44	2.15	< 0.001
senior high school & & technical school& technical secondary school	1.70	1.38	2.11	< 0.001
**Employment status of the head of household (**Ref**:** unemployed and students**)**				
Employed	0.76	0.71	0.82	< 0.001
Retired	0.96	0.84	1.10	0.590
**Medical insurance of the head of household** (Ref: UE-BMI**)**				
UR-BMI	1.68	1.48	1.97	< 0.001
NCMS	1.84	1.59	2.13	< 0.001
IBMIUR	1.64	1.40	1.93	< 0.001
Mixture of schemes	1.43	1.16	1.76	< 0.001
Other types and none	1.39	1.09	1.77	0.001
**Members with chronic diseases** (Ref: ≥2**)**				
0	0.61	0.55	0.67	< 0.001
1	0.87	0.80	0.95	< 0.001
**Inpatient members** (Ref: ≥2**)**				
0	0.25	0.21	0.29	< 0.001
1	0.70	0.60	0.81	< 0.001
**Whether a member went to a clinic** (Ref: No**)**				
Yes	0.76	0.71	0.81	< 0.001
**Members should have been hospitalized but were not** (Ref: No**)**				
Yes	0.63	0.57	0.70	< 0.001
**Members should have seen a doctor but did not** (Ref: No**)**				
Yes	0.81	0.75	0.87	< 0.001

## Discussion

Our study conducted an analysis of poverty and IME incidence and located the characteristics of people with higher IME risk. In the aspect of IME incidence, we found that in 2013, the level of IME in China was 6.3%, which was at a higher level compared with other countries. A recent international study showed that the IME ranged between 1% point and 4% points [20]. In Ethiopia, there were between 1.18 and 1.19% of the total population forced into IME [12]. Kwesiga found that about 4% of the Ugandan population was impoverished by medical spending [21], Other study also found a similar IME incidence in Bangladesh [13]. Through further analysis of the influencing factors of IME, we should focus on the following groups of people.

### Regional level: the joint roles of economic development, health service utilization, and welfare policies in medical impoverishment

Households in underdeveloped central and western regions of China are subject to greater risk of IME. It is noteworthy that while some central provinces are not seriously affected by poverty, some households in these areas have suffered severe IME after paying for medical expenses. Meanwhile, in the western provinces where the poverty rate is high, the rate of IME is not so high. From this survey, the admission rate in the central region (8.0%) is lower than that in the western region (8.6%), but the average hospitalization expenses in the central region (10,254 yuan) is higher than that in the western region (8342 yuan) [18]. The relatively lower utilization of health services in the central region does not contribute to a lighter economic burden. This reflects those regional differences exist in the design of medical insurance schemes, which lead to different patterns of economic burden.

The overall design of national poverty alleviation and medical health insurance reform is crucial. In the early stage of China’s poverty alleviation efforts, the country adopted several measures to address poverty in different regions. Since 1996, the Chinese government has identified poverty alleviation projects that provide financial assistance to 9 provinces in the eastern region and 10 provinces in the western region [22]. From 1995 to 2012, the average annual growth rate of per capita transfer payments in the western region was 20.3%, higher than that in the central region [23]. Meanwhile, according to our calculations, the reimbursement rate in the central region (55.9%) was lower than that in the western region (58.2%) (Additional file 1). Therefore, it is obvious that the economic burden of diseases depends not only on the level of economic development of the region itself, but also on the healthcare needs and service utilization of the people and the design of the medical insurance systems and other welfare systems.

### Household characteristics: poverty and health service utilization are indicative of households with a high incidence of medical impoverishment

We summarized the top five household characteristics associated with the high IME incidence and found that households with inpatient members, those with NCD members, and those at low economic levels suffered the highest incidence of IME. When the determinants of IME are combined together, the incidence of IME was nearly six times that of the national average, especially for poor households that have more than two inpatient members—these had the highest incidence of IME at 35.4%. A family’s economic level and health insurance utilization were found to be indicative of those most vulnerable to IME [15]. Households with a high economic level were better at dealing with the burden of diseases and enjoyed a higher capacity to pay, enabling them to avoid impoverishment [24]. A study in Vietnam also obtained a similar result [25] and remaindered those financial interventions should be targeted at poor households, especially those located in slum areas.

A possible explanation is that, for most developing countries with a large gap between the rich and the poor, although many financial protection measures have been implemented for economically disadvantaged people, ensuring that the poorest are able to benefit more from these reforms should be paid more attention.

### Disease characteristics: chronic diseases lead to medical impoverishment

The diseases that led to IME were mainly chronic diseases [26–28]. Among the top 10 diseases that caused poverty, five were NCDs. Malignant tumors presented the highest risk of IME. China has become a place with a high incidence of malignant tumors, accounting for about 22% of the global incidence of tumor diseases [29]. Although China has launched major disease medical insurance schemes for patients to deal with the economic burden of such diseases since 2012, the compensation level and benefit coverage for major diseases still need to increase [30]. Some tumor diseases, such as benign brain tumor, are not even covered by the major disease medical health insurance schemes, contributing to the high economic burden of residents. Moreover, patients suffering from mental illness shared more OOP expenses due to insufficient reimbursement. Those who enrolled under MIUE for mental illness could only enjoy a 53.9% reimbursement rate compared with those who enrolled for diabetes, who enjoyed 81.5%. Meanwhile, due to the long treatment period for chronic diseases, related indirect expenses such as transportation, nursing, and preventive health care expenses, as well as time lost, were not included in the scope of the reimbursement [31, 32]. All these factors contributed to chronic diseases becoming the risk factor that pushed households into IME.

### Medical insurance scheme level: the inequity existing in different medical insurance schemes

Our results showed that different medical insurance schemes led to different degrees of risk of IME. NCMS enrollment was associated with the highest IME risk, with incidence as high as 9.1%, approximately 4.79 times that of UE-BMI enrollment. The integrated medical insurance scheme was designed to alleviate inequity among different groups; however, at the initial stage, IME was still high at 6.3%. The fundamental reason for this inequity among different medical insurance schemes is the imbalanced design of the financing and reimbursement levels and the benefit package, making it impossible for enrollees to achieve equal access to health services. NCMS, with its relatively lower reimbursement level and insufficient benefit package, provided poor economic protection for residents in China [33]. China’s rural population is large, and it is a group that suffers from major and chronic diseases that lead to significant medical expenses [34]. Due to NCMS’s insufficient compensation level, rural residents have become the main high-risk group for impoverishment. Therefore, it is essential to strengthen the top-level design of the medical insurance system and eliminate the imbalance among insurance schemes to alleviate the risk of IME [35]. Compared to other under protected low- and middle-income countries, the IME level in China seems to be higher, this reflects the extensive use of copayments and insufficient insurance coverage. Therefore, overspending direct medical payment may be the main reason for IME, which is similar to Korea.

### A combined strategy to make poverty alleviation more effective

By the end of 2017, the size of China’s poor population was 30.5 million people [36]. Due to wide-ranging factors such as the aging of the population and social, economic, and geographical imbalances, poverty alleviation has proven to be challenging. To achieve comprehensive poverty alleviation, now is the time to be absolutely precise with regard to the steps taken toward poverty alleviation. How to define the precise target population and how to distribute the country’s resources to help the people at risk of IME have become top priorities.

Due to the multiple vulnerabilities of the poor population, it is no longer meaningful to divide the poor according to the national poverty line. The most vulnerable population can be screened out by accurately identifying specific family characteristics [37]. Having low income is one of the main causes of poverty, but it is not as significant as poor health. If we continue to use income as the only criterion for identifying the poor, the situation will arise wherein right after some people have been lifted out of poverty, other people fall into poverty because of medical expenses. Therefore, this study provides multidimensional criteria to determine vulnerable families, specifically, poor families with at least one inpatient, those with more than two NCD members, those with members suffered from tumor diseases, and those whose heads are unemployed, illiterate, or retired.

Establishing an eligibility screening method that uses multi-dimensional criteria could enable the precise identification of the vulnerable poor who are most at risk of IME. Then, multiple targeted interventions and poverty alleviation strategies can be carried out to deal with diseases. In addition to the three basic medical insurance systems, MFA—the catastrophic disease medical insurance—should also play a complementary role. It is important to abolish the deductible line and upper limit for the poor, increase the reimbursement ratio, and design special preferential medicine purchases for the poor. In this regard, Australia’s approach is worth learning from. Specifically, the Australian government has set a maximum limit for the annual OOP payment of medicines for patients. The upper limit for the normal population (A$ 1317.2) is higher than that for the vulnerable population (A$ 336). Once the medicine payment of a patient (who is vulnerable to poverty) exceeds the upper limit, the excess part is subsidized [38]. We should not only improve the security provided by medical insurance, but also standardize the terms of services of health service providers. In the market economy, doctors may take advantage of information asymmetry between doctors and patients, thereby pursuing personal benefits. As a result, patients would have no choice but to accept all the examinations and treatments prescribed by the hospital, thereby causing accumulated costs of a bottomless pit. Therefore, it is necessary to standardize the treatment plan by adopting diagnosis-related-group payments for inpatient care. It can not only reduce residents’ medical expenses, but also avoid wasting medical funds for poor people.

As the saying goes, “Give a man a fish and you feed him for a day. Teach him how to fish and you feed him for a lifetime.” Analogous to this saying, instead of simply providing economic, medical, and housing assistance to the poor, alleviating poverty is more important when it comes to helping poor people support themselves. Therefore, it is important to provide measures to alleviate industrial poverty and employment poverty among the poor. In this light, it is helpful to organize national leading enterprises to cooperate with poverty-stricken counties and expand the marketing channels of agricultural products, for instance, by selling grains and fruits online. Moreover, increasing the income of labor services can be achieved through job subsidies and loan support programs to encourage poor families to participate in cleaning, road protection, water management, disability assistance, old-age care, and so on [39]. In addition, poverty alleviation requires stronger government leadership to set up new systems that would effectively coordinate various departments in the whole society. Indeed, health poverty alleviation needs to be supplemented by industrial and employment poverty alleviation measures.

### Strengths and limitations of this study

This study focused on factors hinder the pace of China’s alleviation efforts of poverty-stricken population from the perspective of IME. We investigated the incidence of poverty and IME and revealed the features of impoverishment from multi-dimensions using a large sample national survey. We also further explored and identified the precise groups that are vulnerable to IME with 1 or overlapped risk factors. This study also had some limitations. First, it was a cross-sectional study, and the association between impoverishment and predictors could not be obtained. Second, the datasets were obtained via a self-reported questionnaire, the recall bias may exist. Third, we only estimate the impoverishment caused by direct medical expenses and the indirect expenses (e.g., transport, food, accommodation costs, etc.) for patients were excluded, in the future studies, the direct medical expenses and indirect medical-related expenses should be taken into account. Additionally, those can’t afford health service payments were not included in the sample, which might lead to the under-estimation of CHE.

## Conclusion

This study investigated poverty and impoverishment by medical expenses in China and identified the determinants from the aspect of demographic and health service needs and utilization factors. The results highlighted the joint roles of economic development, health service utilization, and welfare policies. Poverty and health service utilization are indicative of households with high incidence of medical impoverishment. Chronic diseases lead to medical impoverishment. The inequity existing in different medical insurance schemes leads to different degrees of IME risk. A combined strategy to precisely target multiple vulnerabilities of poor population should be more effective.

## Supplementary Information

**Additional file 1: Table S1.** Univariate analyses on factors associated with IME. **Figure S1.** Reimbursement expenses and reimbursement rate among different regions.

## Data Availability

Datasets used in this study are available from Centre of Health Statistics and Information, National Health Commission of the People’s Republic of China. The data are not publicly available due to the confidential policy. Requests for the dataset should be directed to Centre of Health Statistics and Information, National Health Commission of the People’s Republic of China. Requests to access these datasets should be directed to the website of National Health Commission of the People’s Republic of China: http://www.nhc.gov.cn/.

## Abbreviations

IME: Impoverishment by medical expense
NCMS: The New Rural Cooperative Medical Scheme
UE-BMI: Urban Employee Basic Medical Insurance
UR-BMI: Urban Resident Basic Medical Insurance
IBMIUR: Integrating Basic Medical Insurance for Urban and Rural Residents
WHO: World Health Organization
NHSS: National Health Service Survey
