# Supplementary material for: What fragile factors hinder the pace of China’s alleviation efforts of the poverty-stricken population? A study from the perspective of impoverishment caused by medical expenses
Source: BMC Health Serv Res. 2022 Jul 29;22:963. doi: 10.1186/s12913-022-08237-2 (PMC9336080; doi:10.1186/s12913-022-08237-2)
Supplement: Supplementary file 1 — Additional file 1: Table S1. Univariate analyses on factors associated with IME. Figure S1. Reimbursement expenses and reimbursement rate among different regions. [file 12913_2022_8237_MOESM1_ESM.docx]

**Table S1** **Univariate analyses on factors associated with IME**

| Characteristic of respondents | Total | Impoverishment | χ2 | P-value |
| --- | --- | --- | --- | --- |
|  | N (%) | N (%) |  |  |
| **Economics quintile** |  |  | 5320.384 | <0.001 |
| Quintile 1 | 18714(20.00) | 2454(13.10) |  |  |
| Quintile 2 | 18714(20.00) | 2530(13.50) |  |  |
| Quintile 3 | 18714(20.00) | 492(2.60) |  |  |
| Quintile 4 | 18714(20.00) | 203(1.10) |  |  |
| Quintile 5 | 18714(20.00) | 190(1.00) |  |  |
| **Household size** |  |  | 380.643 | <0.001 |
| ≤2 | 41633(44.50) | 3318(8.00) |  |  |
| 3-4 | 39289(42.00) | 1844(4.70) |  |  |
| ≥5 | 12648(13.50) | 707(5.60) |  |  |
| **Region** |  |  | 130.367 | <0.001 |
| Eastern | 31201(33.30) | 1578(5.10) |  |  |
| Central | 31186(33.30) | 2037(6.50) |  |  |
| Western | 31183(33.30) | 2254(7.20) |  |  |
| **Location** |  |  | 819.062 | <0.001 |
| Urban | 46798(50.00) | 1874(4.00) |  |  |
| Rural | 46772(50.00) | 3995(8.50) |  |  |
| **Gender** |  |  | 20.267 | <0.001 |
| Male | 69826(74.60) | 4525 (6.50) |  |  |
| Female | 23744(25.40) | 1344 (5.70) |  |  |
| **Marital status of household head** |  |  | 32.138 | <0.001 |
| Married | 78751(84.20) | 4786(6.10) |  |  |
| Others | 14819(15.80) | 1083(7.30) |  |  |
| **Educational level of the head of household** |  |  | 1334.572 | <0.001 |
| Illiterate | 9904(10.60) | 1092(11.00) |  |  |
| Primary school | 26556(28.40) | 2316(8.70) |  |  |
| Junior high school | 33742(36.10) | 1884(5.60) |  |  |
| senior high school & & technical school& technical secondary school | 11800(12.60) | 458(3.90) |  |  |
| Junior college and above | 11568(12.40) | 119(1.00) |  |  |
| **Employment status of the head of household** |  |  | 780.033 | <0.001 |
| Employed | 64026(68.40) | 3856(6.00) |  |  |
| Retired | 16164(17.30) | 530(3.30%) |  |  |
| unemployed and students | 13380(14.30) | 1483(11.10) |  |  |
| **Medical insurance of the head of household** |  |  | 1480.30 | <0.001 |
| UE-BMI | 23919(25.60) | 461(1.90) |  |  |
| UR-BMI | 6847(7.30) | 414(6.00) |  |  |
| NCMS | 43362(46.30) | 3978(9.20) |  |  |
| IBMIUR | 12060(12.90) | 750(6.20) |  |  |
| Mixture of schemes | 4811(5.1%) | 163(3.40) |  |  |
| Other types and none | 2571(2.7%) | 103(4.00) |  |  |
| **Households including members aged above sixty years** |  |  | 513.365 | <0.001 |
| No | 52792(56.40) | 2478(4.70) |  |  |
| Yes | 40778(43.60) | 3391(8.30) |  |  |
| **Households including members aged below five years** |  |  | 22.769 | <0.001 |
| No | 77239(82.50) | 4979(6.40) |  |  |
| Yes | 16331(17.50) | 890(5.40) |  |  |
| **Number of patients with chronic diseases** |  |  | 668.265 | <0.001 |
| 0 | 52793(56.40) | 2374(4.50) |  |  |
| 1 | 30316(32.40) | 2506(8.30) |  |  |
| ≥2 | 10461(11.20) | 989(9.50) |  |  |
| **Number of hospitalized members** |  |  | 1182.948 | <0.001 |
| 0 | 74641(79.80) | 3663(4.90) |  |  |
| 1 | 16795(17.90) | 1918(11.40) |  |  |
| ≥2 | 2134(2.30) | 288(13.50) |  |  |
| **Preferred institution grade for common diseases** |  |  | 362.551 | <0.001 |
| Primary hospital | 75833(81.00) | 5310(7.00) |  |  |
| Non-primary hospital | 17737(19.00) | 559(3.20) |  |  |
| **Whether there is a member go to clinic** |  |  | 292.215 | <0.001 |
| No | 75006(80.20) | 4199(5.60) |  |  |
| Yes | 18564(19.80) | 1670(9.00) |  |  |
| **Members should be hospitalized but not** |  |  | 426.175 | <0.001 |
| No | 89516(95.70) | 5303(5.90) |  |  |
| Yes | 4054(4.30) | 566(14.00) |  |  |
| **Members should see a doctor but not** |  |  | 161.240 | <0.001 |
| No | 66440(71.00) | 3740(5.60) |  |  |
| Yes | 27130(29.00) | 2129(7.80) |  |  |

**Figure S1** Reimbursement expenses and reimbursement rate among different regions
